# Case report: Xenotransfusion of canine lyophilized platelets for hemostasis in a feline patient with post-operative abdominal hemorrhage

**DOI:** 10.3389/fvets.2023.1113846

**Published:** 2023-02-07

**Authors:** Elizabeth Mannucci, April Blong, Chelsea Zorn, Rebecca Walton

**Affiliations:** ^1^Emergency and Critical Care Department, VCA West Los Angeles Animal Hospital, Los Angeles, CA, United States; ^2^Department of Clinical Sciences, Iowa State University, Ames, IA, United States

**Keywords:** hemorrhage, platelets, transfusions, thrombocytopenia, xenotransfusion

## Abstract

**Objective:**

To describe the management of post-operative abdominal hemorrhage with a xenotransfusion of canine lyophilized platelets in a feline patient.

**Case summary:**

A 9-year-old male castrated domestic shorthair presented for a spontaneous hemoabdomen secondary to hepatic amyloidosis. Clinically significant hemorrhage occurred in the perioperative and post-operative period and the patient received a massive transfusion and anti-fibrinolytic therapy in combination with a xenotransfusion of canine lyophilized platelets at 0.9 × 10^9^ particles/kg and recombinant human factor VIIa (rhFVIIa). The combination of these interventions decreased transfusion requirements in this patient and the xenotransfusion was well tolerated with no acute or immediate transfusion reactions noted.

**New or unique information provided:**

This case report describes the xenotransfusion of canine lyophilized platelets in a feline patient with severe, non-compressible abdominal hemorrhage.

## 1. Introduction

Platelet transfusions are indicated in the management of uncontrollable and life-threatening hemorrhage or with severe thrombocytopenia or thrombocytopathia (1). Massive transfusion protocols typically include platelet and plasma products, in combination with red blood cells, to reduce the risk of coagulopathy and improve outcome (2, 3). Additionally, prophylactic perioperative platelet transfusions are indicated in patients with a known bleeding tendency, hereditary thrombocytopathia, or severe thrombocytopenia (<10–20 × 10^9^/L) (1, 4). In these patients, prophylactic platelet transfusions have been shown to reduce transfusion requirements during surgical procedures.

Unfortunately, platelet transfusions pose a significant clinical challenge in veterinary medicine due to a lack of readily available whole blood donors, and the short shelf-life of platelet-rich plasma and fresh platelet concentrate (2). Generally, the development of feline platelet products is restricted by the small volume of feline fresh whole blood units. Therefore, cats in need of platelets typically receive fresh whole blood as no commercially available feline platelet products exist (5). Alternative platelet products, including canine lyophilized platelets, have been noted to be effective in the management of hemorrhage in thrombocytopenic dogs, however, to the authors' knowledge, there have been no reports of xenotransfusions of platelet products in cats (6). This report describes a xenotransfusion of canine lyophilized platelets to a cat with severe, non-compressible hemorrhage secondary to hepatic amyloidosis.

## 2. Case summary

**Figure 1:**
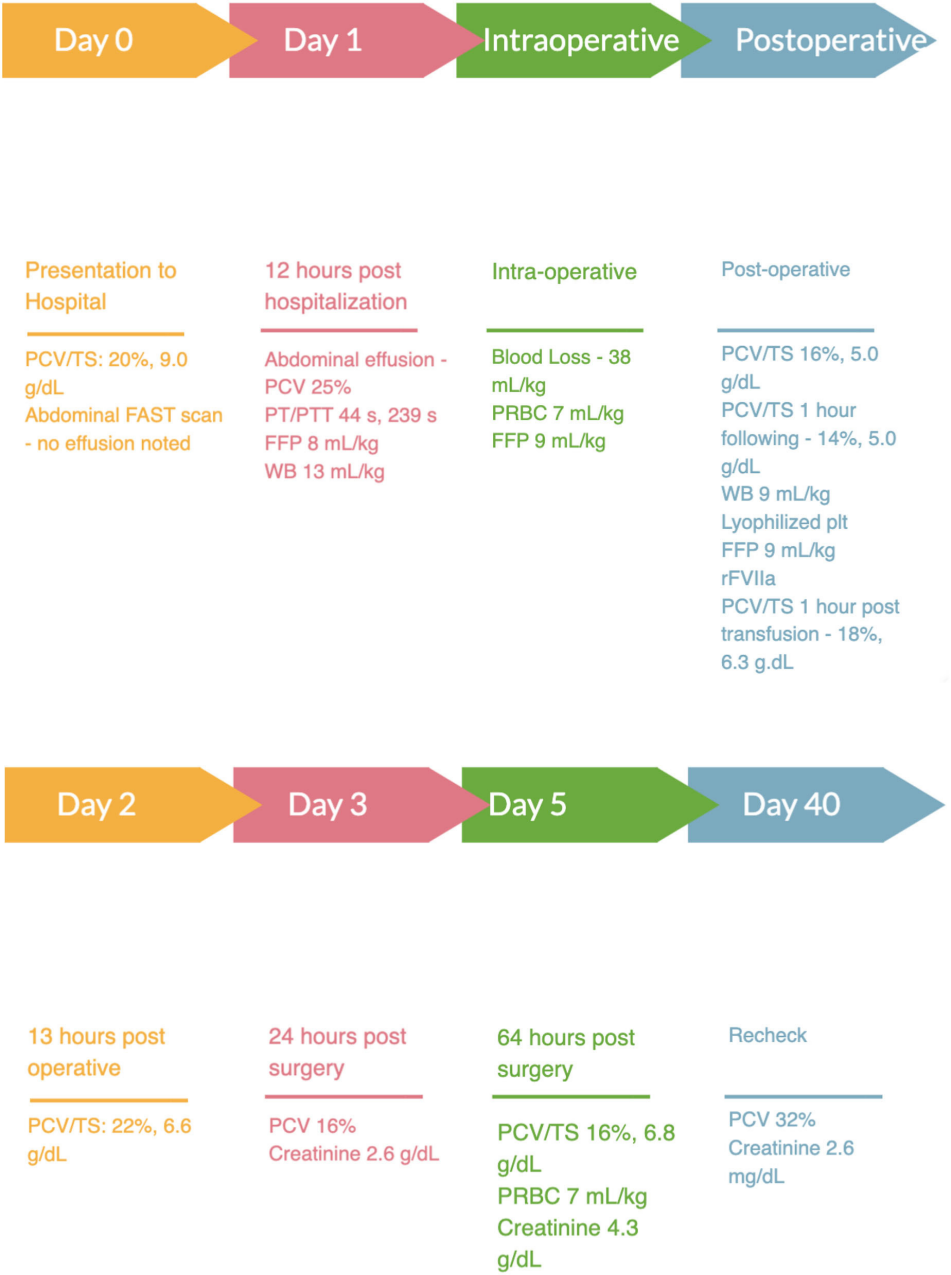
Timeline of hospitalization. PCV, packed cell volume; TS, total solids; FFP, fresh frozen plasma; WB, whole blood; PRBC, packed red blood cells; rFVIIa, recombinant factor VII.

A 9-year-old male castrated domestic shorthair weighing 5 kg was presented to a university teaching hospital for a 1-day history of lethargy and anorexia. The cat had a 5-year history of chronic kidney disease, International Renal Interest Society stage 3 (non-hypertensive, non-proteinuric), with a baseline creatinine of 2.9 g/dl, 256 μmol/L (RI, 0.5–1.8 mg/dl; 35–124 μmol/L) 1 month prior to presentation. On presentation, the cat's vital signs were within normal limits, temperature 100.8 F, heart rate 210 bpm and respiratory rate of 50 bpm. A grade II/VI left systolic parasternal was noted with no other abnormalities appreciated on presenting physical exam. Point-of-care bloodwork was performed and revealed an anemia with a packed cell volume (PCV) of 20% (RI, 35%−45%) and a total solid (TS) of 9.0 g/dl (RI, 5.2–8.2 g/dl). A venous blood gas was performed which revealed a mild metabolic acidosis (pH: 7.270; RI, 7.31–7.46; base excess: −10.3; RI −4 to 4) with a hyperlactatemia (3.64 mmol/L; RI, 0–2.5 mmol/L). Focused assessment with sonography for trauma, tracking and triage (FAST) scan was negative for abdominal or thoracic effusion at presentation. The cat was hospitalized on intravenous fluids with a balanced isotonic crystalloid at 85 ml/kg/day and ondansetron^1^ (1 mg/kg IV q8h). Recheck lactate 8 h following presentation was 2.6 mmol/L (RI, 0–2.5 mmol/L). Abdominal radiographs performed 12 h after presentation revealed moderate peritoneal effusion and mild generalized hepatomegaly. Repeat FAST scan 12 h following presentation revealed peritoneal effusion with a fluid score of 4/4 and sampling was confirmatory for a hemoabdomen with a PCV of 25%. Coagulation times pre-operatively were prolonged [prothrombin time (PT): 44 s, RI 11–17 s; partial thromboplastin time (PTT): 239 s, RI 72–102 s] so the patient was administered a type specific fresh frozen plasma (FFP; 8 ml/kg IV) and type specific fresh whole blood (WB; 13 ml/kg IV) in the perioperative period (Figure 1). Due to the noted hepatomegaly and lack of exposure to rodenticide or other toxins the cat was taken to exploratory laparotomy. The cat was induced under general anesthesia and an exploratory laparotomy found a bleeding mass involving both the left medial and lateral liver lobes. The affected liver lobes were ligated, resected, and removed for histopathology. The remainder of the liver parenchyma was grossly pale, tan, and friable. Multifocal hemorrhage was noted with minimal manipulation and two topical hemostatic agents were applied^2^,^3^ to minimize further bleeding. During anesthesia, indirect mean arterial pressure (MAP) was 60 mmHg and a dopamine^4^/dobutamine^5^ constant rate infusion (CRI; 3–10 mcg/kg/min IV) was initiated to maintain a MAP of 65–80 mmHg. A type specific packed red blood cell (PRBC) transfusion (7 ml/kg IV) was administered intraoperatively as well as a second dose of type specific FFP (9 ml/kg IV), ~90 min after induction of anesthesia. The cat additionally received a total 500 mg of calcium gluconate IV and 1.5 mEq of magnesium sulfate following completion of the PRBC and FFP. Overall, the patient was estimated to lose ~38 ml/kg of blood during the procedure. An initial dose of aminocaproic acid^6^ (100 mg/kg IV) was administered during surgery. Upon recovery, the patient was hypotensive, the CRI of dopamine/dobutamine was discontinued and a norepinephrine^7^ CRI (0.5–1.0 mcg/kg/min) was initiated. The PCV immediately post-operatively was 16% (RI, 35%−45%) with a total solid of 5.0 g/dl (RI, 5.2–8.2 g/dl) and a lactate of 1.5 mmol/L (RI, 0–2.5 mmol/L). The norepinephrine CRI was continued following recovery. One hour following recovery, the patient was noted to be progressively anemic with a PCV of 14% (RI, 35%−45%) and a total solid of 5.0 g/dl (RI, 5.2–8.2 g/dl) with progressive peritoneal fluid accumulation. Due to the combination of progressive anemia, peritoneal effusion progression, cardiovascular instability, and multifocal hemorrhage noted in surgery the patient was given another type specific fresh WB transfusion (9 ml/kg IV), a xenotransfusion of canine lyophilized platelets^8^ (0.9 × 10^9^ particles/kg) and an additional FFP transfusion (9 ml/kg IV) and a 0.45 mg (0.09 mg/kg IV) dose of rFVIIa.^9^ All transfusions were well tolerated with no immediate transfusion reactions noted. Following administration of canine lyophilized platelets, rFVIIa and completion of the fresh WB transfusion, the patient's PCV was 18% (RI, 35%−45%) and TS were 6.3 g/dl (RI, 5.2–8.2 g/dl). Additionally, the patient's hypotension resolved, and norepinephrine was discontinued. Post-operatively the patient was managed with ondansetron 1 mg/kg IV q8h, maropitant^10^ 1 mg/kg IV q24, aminocaproic acid^6^ 30 mg/kg IV q4h, fentanyl^11^ 3 mcg/kg/h, ketamine^12^ 0.3 mg/kg/h and a balanced isotonic crystalloid fluid at a rate of 85 ml/kg/day. Thirteen hours post lyophilized platelet and rFVIIa administration the patient's PCV was 22% (RI, 35%−45%) with a TS of 6.6 g/dl (RI, 5.2–8.2 g/dl) and the abdominal effusion was noted to be subjectively static. The day following surgery the patient's PCV was 16% (RI, 35%−45%) and remained static for the first 3 days post-operatively. Approximately 64 h following recovery from surgery the patient's PCV was 13% (RI, 35%−45%) and TS 6.8 (RI, 5.2–8.2 g/dl) so the patient was administered a cross-match compatible PRBC transfusion (7 ml/kg IV) over 4 h. Histopathology of liver lobes revealed microvascular dysplasia with portal venule hypoplasia and amyloidosis.

Four days post-operatively, the cat developed an acute kidney injury (AKI) with an increase in creatinine to 4.3 mg/dl; 380 μmol/L (RI, 0.8–2.1 mg/dl; 35–124 μmol/L) during intravenous fluid taper, from 2.6 mg/dl; 256 μmol/L (RI, 0.5–1.8 mg/dl; 35–124 μmol/L), the day prior. Intravascular fluid support was continued, and the acute kidney injury resolved over the following days. The cat was discharged from the hospital seven days following presentation with a creatinine of 2.9 mg/dl; 256 μmol/L (RI, 0.8–2.1 mg/dl; 35–124 μmol/L). At last follow up, 34 days following discharge, the patient's clinical status had returned to normal and creatinine returned to the previous baseline of 2.6 mg/dl; 256 μmol/L (RI, 0.8–2.1 mg/dl; 35–124 μmol/L) and the PCV was improved to 32% (RI, 35%−45%).

## 3. Discussion

Platelet products are not readily accessible due to donor availability and storage challenges (2). Canine platelet products have been recently developed, including cryopreserved and lyophilized platelets. Canine cryopreserved platelets are commercially available and canine lyophilized platelets have recently been evaluated as an alternative product due to ease of storage, administration, and prolonged storage time (2, 6). Both canine platelet products have been utilized in dogs to manage hemorrhage secondary to thrombocytopenia and in controlling non-compressible, life-threatening hemorrhage (2, 4).

Xenotransfusion, the transfusion of blood from one species to another, has historically been performed in cats due to the limited availability of donors and products (7, 8). Xenotransfusion is often implemented during short-term stabilization in cases of absent blood product availability, including lack of platelet products, or blood type incompatibilities (7). While a potentially life-saving intervention, the benefits of xenotransfusion are short-lived compared to allotransfusions due to the development of antibodies and the premature destruction of donor cells (7). Xenotransfusions are additionally associated with a high risk of transfusion reactions (1, 9). The incidence of hemolytic transfusion reactions in cats receiving canine packed red blood cells has been reported as high as 64%, occurring within 2–4 days of xenotransfusion, suggesting rapid antibody development (2, 10).

No previous reports exist regarding xenotransfusion of platelet products in cats. Both cryopreserved and lyophilized platelets have been utilized in dogs and transfusion reactions in dogs receiving platelet concentrates is reportedly low, with mild signs such as vomiting, pyrexia, tachycardia and facial swelling occurring in ~14% of cases (2, 4). No transfusion reactions have been reported in dogs receiving lyophilized platelets (6). The cat in this case report received a canine lyophilized platelet transfusion in addition to multiple feline blood products and rhFVII. The combination of blood products, including lyophilized platelets, resulted in cessation of non-compressible abdominal hemorrhage secondary to hepatic amyloidosis. The cat additionally did not develop any evidence of immediate transfusion reactions secondary to this xenotransfusion. The cat did develop an AKI 4 days post-operative, which cannot be determined if this may have been related to the xenotransfusion or other causes.

Due to the number and timing of interventions performed, it is not possible to determine how much effect, if any, the canine lyophilized platelets contributed to cessation of bleeding. However, this case demonstrated the use of canine lyophilized platelets in a cat with severe hemorrhage.

## Data availability statement

The original contributions presented in the study are included in the article/supplementary material, further inquiries can be directed to the corresponding author.

## Author contributions

EM, AB, CZ, and RW were all involved in writing of the manuscript. All authors contributed to the article and approved the submitted version.

## Footnotes

^1^Ondansetron, Accord Healthcare, Inc, Durham, NC.

^2^HemaBlock, HemaBlock, LLC for Vet Brands International, Inc, Miramar, FL.

^3^Surgicel, Johnson and Johnson (Ethicon), Irvine, CA.

^4^Dopamine, Hospira, Inc., Lake Forest, IL.

^5^Dobutamine, Hospira, Inc., Lake Forest, IL.

^6^Aminocaproic acid, Hospira, Inc., Lake Forest, IL.

^7^Norepinephrine, Baxter, Deerfield, IL.

^8^Lyophilized platelets (StablePlate RX), BodeVet, Rockville, MD.

^9^Recombinant human factor seven (NovoSeven RT), Novo Nordisk, Plainsboro, NJ.

^10^Maropitant Cerenia^®^, Pfizer, New York, NY.

^11^Fentanyl Hospira, Lake Forrest, IL (Pfizer).

^12^Ketamine Pfizer, New York, NY.
